# Characterisation of a novel cardiac phenotype in patients with GFPT1 or DPAGT1 mutations

**DOI:** 10.1186/1532-429X-16-S1-P332

**Published:** 2014-01-16

**Authors:** Andrew Lewis, Sarah Finlayson, Masliza Mahmod, Theodoros D Karamitsos, Sairia Dass, Houman Ashrafian, Jane M Francis, Hugh Watkins, David Beeson, Jacqueline Palace, Stefan Neubauer

**Affiliations:** 1 University of Oxford, Oxford, UK

## Background

Mutations in the GFPT1 and DPAGT1 genes, which encode enzymes associated with roles in protein N-linked glycosylation, have been recently identified in a rare subgroup of patients with congenital myasthenic syndromes (CMSs). These mutations are inherited in an autosomal recessive pattern, and the mechanism of impaired neuromuscular transmission may be acetylcholine receptor (AChR) deficiency due to impaired (AChR) subunit glycosylation. Aberrant protein glycosylation is also implicated in the development of severe cardiomyopathies in the congenital disorders of glycosylation, although the mechanisms responsible for cardiac involvement are unknown. We investigated whether patients with CMS and GFPT1 or DPAGT1 mutations also had evidence of a cardiac phenotype.

## Methods

We performed comprehensive cardiovascular magnetic resonance (CMR) imaging at 1.5T (Avanto, Siemens), 31P spectroscopy at 3T (Tim Trio, Siemens) and echocardiography to evaluate cardiac structure and function in patients with GFPT1 (n = 2) and DPAGT1 (n = 2) mutations. The mean age of the participants was 45 (range 25-57) and two were male.

## Results

**Figure 1:**
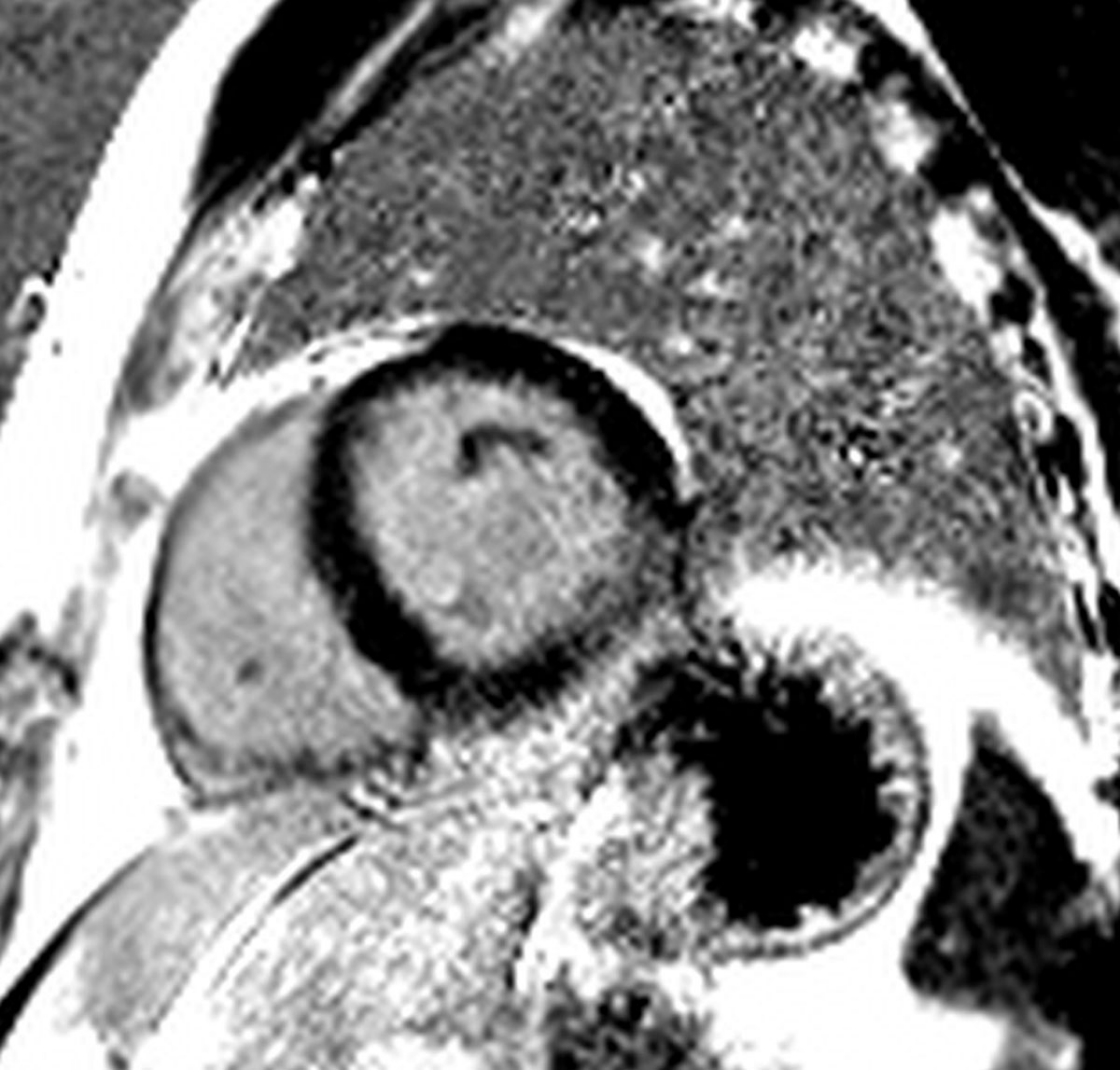
**Representative electrocardiograph demonstrating repolarization abnormalities with inversion of T-waves in inferolateral leads and deep S waves**.

Electrocardiography was abnormal in all, with abnormal repolarisation and deep S waves (n = 3) or marked left ventricular hypertrophy by voltage criteria (n = 1). Despite normal biventricular volumes and systolic function, GFPT1/DPAGT1 patients demonstrated late gadolinium enhancement suggestive of myocardial fibrosis (n = 4, mean proportion of enhanced myocardium > 5 SD above individual reference regions 3.2% +/-1.6, Figure 1), impaired energetics (n = 2) and diastolic dysfunction (n = 3). No patient had symptoms attributable to cardiovascular disease on structured interview.

## Conclusions

Patients with GFPT1 or DPAGT1 mutations demonstrate a cardiac phenotype including abnormal electrocardiography, myocardial fibrosis, diastolic dysfunction and impaired energetics, despite normal systolic function. These findings may reflect incipient cardiomyopathy due to aberrant cardiac glycoprotein function. The reason for the milder phenotype than is seen many congenital disorders of glycosylation may be greater residual enzyme function as a consequence of a less pathogenic mutation. This study highlights the utility of CMR for the assessment of rare cardiac phenotypes and reinforces the need for cardiac surveillance in patients with undefined or uncharacterised neuromuscular disorders due to glycosylation pathway defects.

## Funding

British Heart Foundation.

**Table 1:** 

	Patient 1	Patient 2	Patient 3	Patient 4	Reference range
Age/years	57	58	38	25	

Gender	Female	Female	Male	Male	

Mutation	DPAGT1	DPAGT1	GFPT1	GFPT1	

Nucleotide change	c.349G > A c.699dup	c.478G > A c.574G > A	c.44C > T c.1486C > T	c.1154G > A c.1301G > A	

Effect on protein	p.Val117Ile p.Thr234Hisfs*116	p.Gly160Ser p.Gly192Ser	p.Thr15Met p.Arg496Trp	p.Arg385His p.Arg434His	

LVEDV/ml	104	142	124	144	102-218 ml (male) 83-187 (female

LVESV/ml	29	53	41	46	18-82 ml (male) 18-66 ml (female)

LVEF	72	63	67	68	57-81% (male and female)

RVEF	75	64	66	70	47-71% (male) 53-73% (female)

LV mass/g	120	115	134	124	81-165 g (male) 42-150 g (female)

LGE regions	Anterior septum and inferior LV/RV junction	Basal inferolateral wall	Basal inferolateral wall	Inferior and inferolateral wall	

LGE > 5SD/%	1.6	2.3	5.1	3.9	

Stress perfusion CMR (visual assessment)	Normal	Not performed	Normal	Normal	

PCr/ATP ratio	1.19	1.01	2.23	2.25	

Diastolic function	Grade 1 dysfunction	Grade 2 dysfunction	Grade 1 dysfunction	Normal	

Baseline characteristics and CMR and echo indices including left ventricular end diastolic volume (LVEDV), left ventricular end systolic volume (LVESV), left ventricular ejection fraction (LVEF), right ventricular ejection fraction (RVEF), late gadolinium enhancement (LGE) and phosphocreatine to adenosine triphosphate ratio (PCrR/ATP).

